# Plasma and Serum Proteins Bound to Nanoceria: Insights into Pathways by which Nanoceria may Exert Its Beneficial and Deleterious Effects *In Vivo*

**Published:** 2020-07-17

**Authors:** Allan D Butterfield, Binghui Wang, Peng Wu, Sarita S. Hardas, Jason M. Unrine, Eric A. Grulke, Jian Cai, Jon B. Klein, William M. Pierce, Robert A. Yokel, Rukhsana Sultana

**Affiliations:** 1 Department of Chemistry, University of Kentucky, Lexington, KY 40506, USA;; 2 Department of Chemical and Materials Engineering, University of Kentucky, Lexington, KY 40506, USA;; 3 Department of Plant and Soil Sciences, University of Kentucky, Lexington, KY 40506, USA;; 4 Department of Pharmacology & Toxicology, University of Louisville School of Medicine, Louisville, KY, 40202, USA;; 5 Department of Medicine, University of Louisville School of Medicine, Louisville, KY, 40202, USA;; 6 Department of Pharmaceutical Sciences, University of Kentucky, Lexington, KY 40536-0596, USA

**Keywords:** Nanoceria, Plasma, Serum, Proteomics, Protein corona

## Abstract

Nanoceria (CeO_2_, cerium oxide nanoparticles) is proposed as a therapeutic for multiple disorders. In blood, nanoceria becomes protein-coated, changing its surface properties to yield a different presentation to cells. There is little information on the interaction of nanoceria with blood proteins. The current study is the first to report the proteomics identification of plasma and serum proteins adsorbed to nanoceria. The results identify a number of plasma and serum proteins interacting with nanoceria, proteins whose normal activities regulate numerous cell functions: antioxidant/detoxification, energy regulation, lipoproteins, signaling, complement, immune function, coagulation, iron homeostasis, proteolysis, inflammation, protein folding, protease inhibition, adhesion, protein/RNA degradation, and hormonal. The principal implications of this study are: 1) The protein corona may positively or negatively affect nanoceria cellular uptake, subsequent organ bioprocessing, and effects; and 2) Nanoceria adsorption may alter protein structure and function, including pro- and inflammatory effects. Consequently, prior to their use as therapeutic agents, better understanding of the effects of nanoceria protein coating is warranted.

## INTRODUCTION

Nanoceria (aka: ceria (CeO_2_) nanoparticles) have extensive uses as an industrial abrasive in chemical mechanical polishing/planarization, a catalyst in diesel fuel, and are being developed for use in fuel cells and batteries [1–5]. While there is little indication of nanoceria-induced adverse environmental effects at current exposure rates from use as a fuel catalyst [6], it is critical to understand its interaction with mammalian components. Nanoceria have anti-inflammatory and pro-/antioxidant activity [2,7–10]. Their antioxidant properties are based on its ability to reversibly bind oxygen and cycle between the Ce^3+^ (reduced) and Ce^4+^ (oxidized) forms at its surface [2,8,11]. Further, studies showed that nanoceria can protect cells against reactive oxygen species (ROS) such as superoxide radical anion and hydrogen peroxide, thereby suggesting it might have SOD- and catalase-mimicking activity [3,9,12,13]. In contrast, there are reports of nanoceria-induced pro-oxidant effects including lipid peroxidation, elevation of cytokines, and GSH depletion [10,14–16].

Nanoceria has been suggested for potential use in nanomedicine for the treatment of many conditions, including ischemia; diabetic cardiomyopathy; gastric, ovarian, pancreatic, and breast cancer; macular degeneration; and Alzheimer disease, among other disorders [3,17]. For most therapeutic purposes, nanoceria will need to be administered via systemic or pulmonary routes due to its very limited oral bioavailability [10]. Once in the blood, nanoparticles become coated by proteins to form a protein corona, which changes their surface properties, and “what the cell sees” [18–20]. However, there is limited information on nanoceria interaction with blood proteins. It has been shown that nanoceria adsorbs proteins from serum [21,22], that net negatively charged albumin and fibrinogen and net positively charged lysozyme can adsorb onto nanoceria surfaces [23–29], and that nanoceria interacts with immunoglobulins [24,29]. However, these studies do not provide insight into the blood proteins that adsorb onto nanoceria surfaces *in vivo*. Proteins adsorbed by nanoparticles appear to be unique to each nanoparticle, creating a “fingerprint for nanoparticle identification” [22,30]. Different proteins in plasma/serum could influence the resultant biological properties of nanoceria. Reportedly, nanomaterials adsorb different proteins from plasma vs. serum [31] and nanoceria’s surface coating can affect its cell interaction [32]. Hence, it is critical to understand the interaction of nanoceria with blood proteins.

To our knowledge, this is the first *in vitro* study that used a proteomics approach to identify the proteins from serum and plasma that adsorbed to nanoceria. The results indicate that a number of proteins from plasma and serum interact with nanoceria. These proteins in their normal state are known to play important roles in regulating numerous cell functions, hence binding of ceria nanoparticles to proteins could affect their functions and thereby could have detrimental effect on normal cellular and physiological processes.

## MATERIALS AND METHODS

All materials used were purchased from Sigma-Aldrich unless stated otherwise.

### Nanoceria synthesis and characterization

Nanoceria synthesis and characterization were described [28]. Hydrodynamic diameter of the citrate-coated nanoceria before plasma or serum exposure was determined from one observation, and after plasma or serum exposure on two observations each. Zeta potential was determined at physiological pH (for the citrate-coated nanoceria and in rat plasma and serum) from four determinations.

### Plasma and serum preparation

Animal work was approved by the University of Kentucky Institutional Animal Care and Use Committee. Five male Sprague-Dawley rats, weighing 328 ± 21 g (mean ± SD), obtained from Harlan, Indianapolis, IN, were deeply anesthetized to obtain whole blood via cardiac puncture of the left ventricle with a 1 cc syringe. Blood was transferred to sterile 500 μl EDTA tubes and immediately centrifuged at 2500 rpm for 5 min to obtain plasma. Plasma was distributed into aliquots, frozen in liquid nitrogen, and stored at −80°C until subsequent analysis. For serum preparation, blood was delivered to sterile tubes containing no anticoagulants. Upon clotting, the tubes were centrifuged and the serum distributed into aliquots, frozen in liquid nitrogen, and stored at −80°C until further investigation.

### Nanoceria incubation with plasma or serum

**Figure 1:**
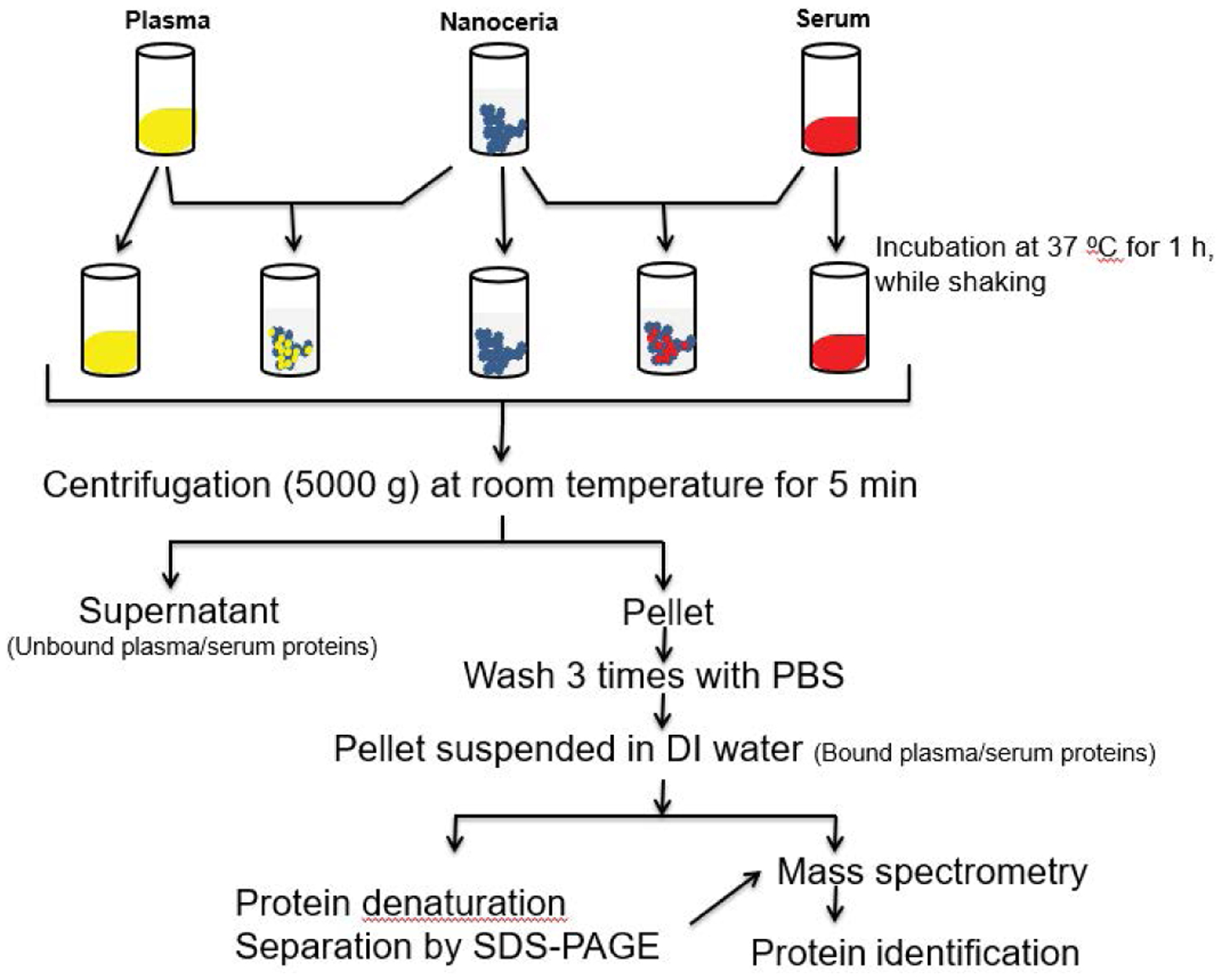
The experimental design for identification of the plasma and serum proteins bound to nanoceria.

The protocol for nanoceria incubation with plasma or serum and identification of associated proteins is shown in Figure 1. Briefly, to 50 μl of 5 weight percent, citrate-coated nanoceria aqueous dispersion, 450 μl of plasma or serum were added. Samples were incubated at 37°C for 1 h with shaking at 500 rpm. Nanoceria was pelleted at 5000 × g for 5 min. The supernatant was collected and stored at −80°C as the unbound protein sample for SDS-PAGE analysis. The pellet was washed three times in 0.5 ml PBS to remove the unbound or loosely bound proteins then resuspended in 100 μl of distilled water as a sample for the analysis of nanoceria-bound proteins. Four replicates were conducted with each blood derivative.

### SDS-PAGE analysis

The bound proteins were removed from the nanoceria by adding SDS-PAGE loading buffer to 20 μl of the suspended pellet and boiling the samples for 5 min at 100°C. The proteins were separated by 12% SDS-PAGE. The gels were fixed and stained overnight in SYPRO^®^ ruby (Bio-Rad).

### Cerium quantitation in SDS-PAGE gel

Selected regions of the SDS-PAGE gel were cut out for cerium quantitation. Samples were obtained from the tops and centers (at ^~^80 kDa) of lanes 5 and 8 of below figure. Spike recovery of a sample from the middle of lane 5 showed 95% recovery. Samples were digested in a 2:1 mixture of trace metal grade HNO_3_ and concentrated H_2_O_2_. The resulting digestates were analyzed by inductively coupled plasma mass spectrometry (ICP-MS; Agilent 7500cx, Santa Clara, CA) using external Ce standards and Tb as an internal standard. Duplicates, reagent blanks, and spike recovery samples were included in each analytical batch. These methods have been described in more detail in a previous publication [33].

### Protein preparation for mass spectrometry

The DI-suspended plasma- (or serum-) coated nanoceria were centrifuged at 5000 × g for 1 min to pellet the particles, the supernatant decanted and the pellets dried by SpeedVac^®^ and analyzed as particle samples. To each sample, 25 μl of 8 M urea/2 mM dithiothreitol (DTT)/50 mM ammonium bicarbonate (NH_4_HCO_3_) were added and the samples incubated at 65°C for 30 min (samples were agitated twice to keep the nanoparticles suspended). The samples were cooled to room temperature, followed by addition of 25 μl of 50 mM iodoacetic acid (IAA) and incubation in the dark for 15 min. The samples were then diluted with 170 μl 50 mM NH_4_HCO_3_ and incubated overnight at 37°C with 4 μl of trypsin (Promega, modified trypsin, frozen, about 0.5 μg/μl). Following overnight protein digestion, 100 μl of 1% formic acid (FA) were added to the samples, which were then desalted with C18 spin columns (The Nest Group, P/N SUM SS18V). Briefly, for desalting, samples were loaded onto the columns, washed 3 times with 100 μl 5% acetonitrile (ACN)/0.1% FA, and eluted twice with 100 mL 50% ACN/0.1% FA followed by concentration to about 5 to 10 mL by SpeedVac^®^. The samples were analyzed by MS/MS.

### MS/MS-based protein identification

#### Several sample types were compared.

Plasma or serum alone was compared to proteins from nanoceria that had been incubated with plasma or serum, as shown in Figure 1. The proteomics methods employed were described [34–36]. Briefly:

#### Image analysis:

Band intensities from SYPRO^®^ Ruby-stained 1D-gel images of samples were compared, and protein bands showing greater staining intensities of proteins from nanoceria exposed to plasma or serum were selected for analyses.

#### In-gel trypsin digestion/peptide extraction:

Protein bands from plasma or serum identified as significantly altered were excised from 1D-gels and transferred to individual Eppendorf microcentrifuge tubes for trypsin digestion as described [37]. In brief, DTT and IAA were used to break and cap disulfide bonds and the gel plug was incubated overnight at 37°C with shaking in modified trypsin solution. Salts and contaminants were removed from the tryptic peptide solutions using C18 ZipTips^®^. Tryptic peptide solutions were reconstituted in 10 μL of a 5% ACN/0.1% FA solution and stored at −80°C until MS/MS analysis.

#### NanoLC-MS with data dependent scan:

Tryptic peptide solutions were analyzed by a nanoAcquity (Waters, Milford, MA)-LTQ Orbitrap XL (Thermo Scientific, San Jose, CA) platform with a data dependent scan mode. An in-house packed capillary column (0.1 × 130 mm packed with 3.6 μm, 200 Å XB-C18) was used for separation using 0.1% FA and ACN/0.1% FA at 200 nl/min. The spectra obtained by MS were measured by the orbitrap at 30,000 resolution and the MS/MS spectra of the six most intense parent ions in the MS scan were acquired by the orbitrap at 7,500 resolution.

### Data analysis and statistics

Nanoceria hydrodynamic sizes before and after plasma and serum exposure were compared by the Kolmogorov– Smirnov test. The Proteome Discoverer v1.4 version of the Swiss-Prot database by SEQUEST (Thermo Scientific) was used to interrogate the MS data files of each sample. At least two high-confidence peptide matches were used for protein identification where the false discovery rate was <1%. Proteins that were matched with the same peptides were reported as one protein group. Protein data reported from these analyses include: the Swiss-Prot accession number, the percentage of the protein sequence identified by matching peptides, the number of peptide sequences identified by the MS/MS analysis, the confidence score of the protein, the expected molecular weight, and predicted isoelectric point.

Protein contents determined from SDS-PAGE gels were compared by Student’s *t-test*. Statistical significance was accepted at p<0.05.

## RESULTS AND DISCUSSION

**Figure 2:**
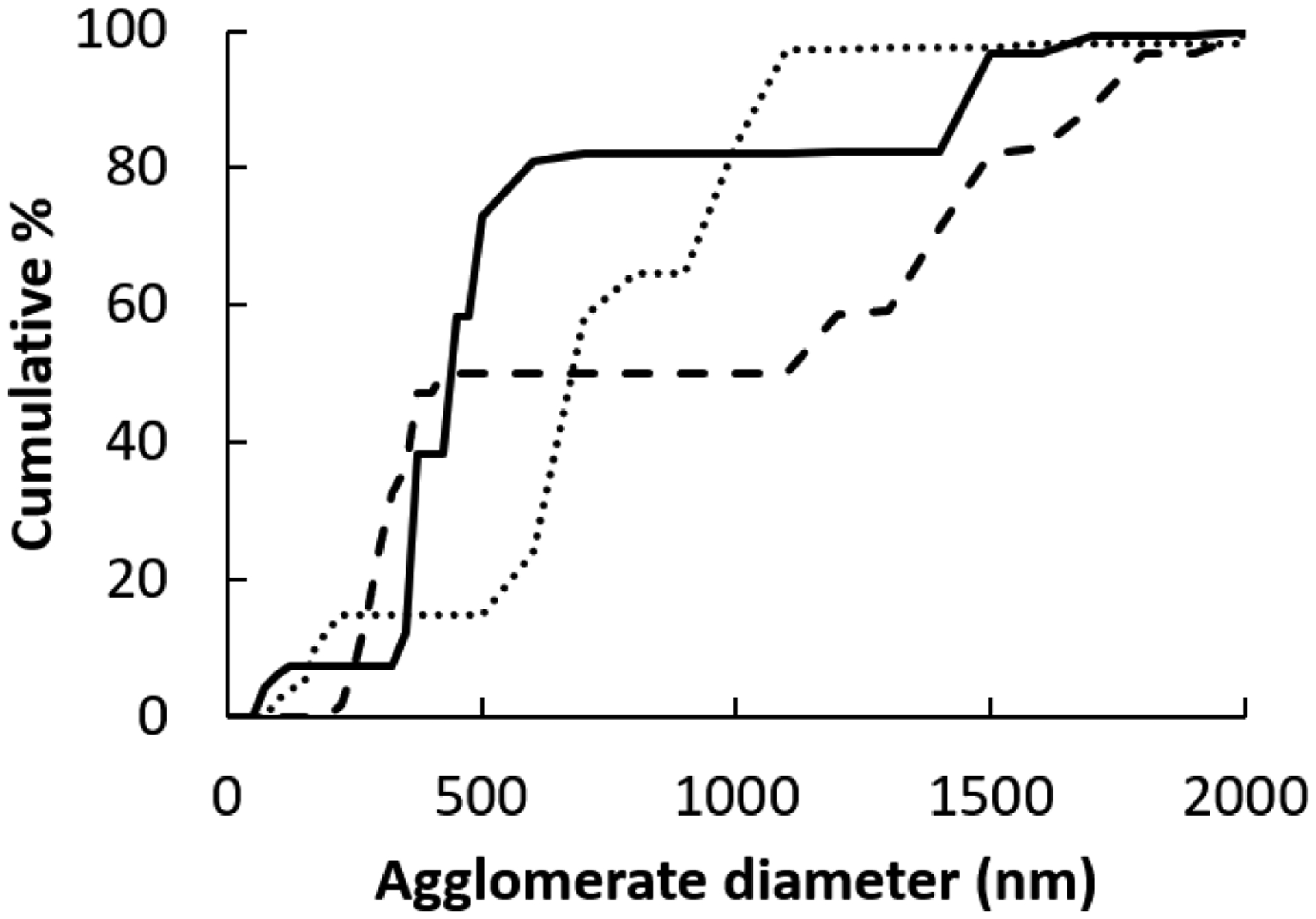
Nanoceria hydrodynamic diameter before and after rat plasma or serumexposure. Solid line is before procedure exposure, dotted line after plasma, and dashed line after serum exposure.

**Figure 3:**
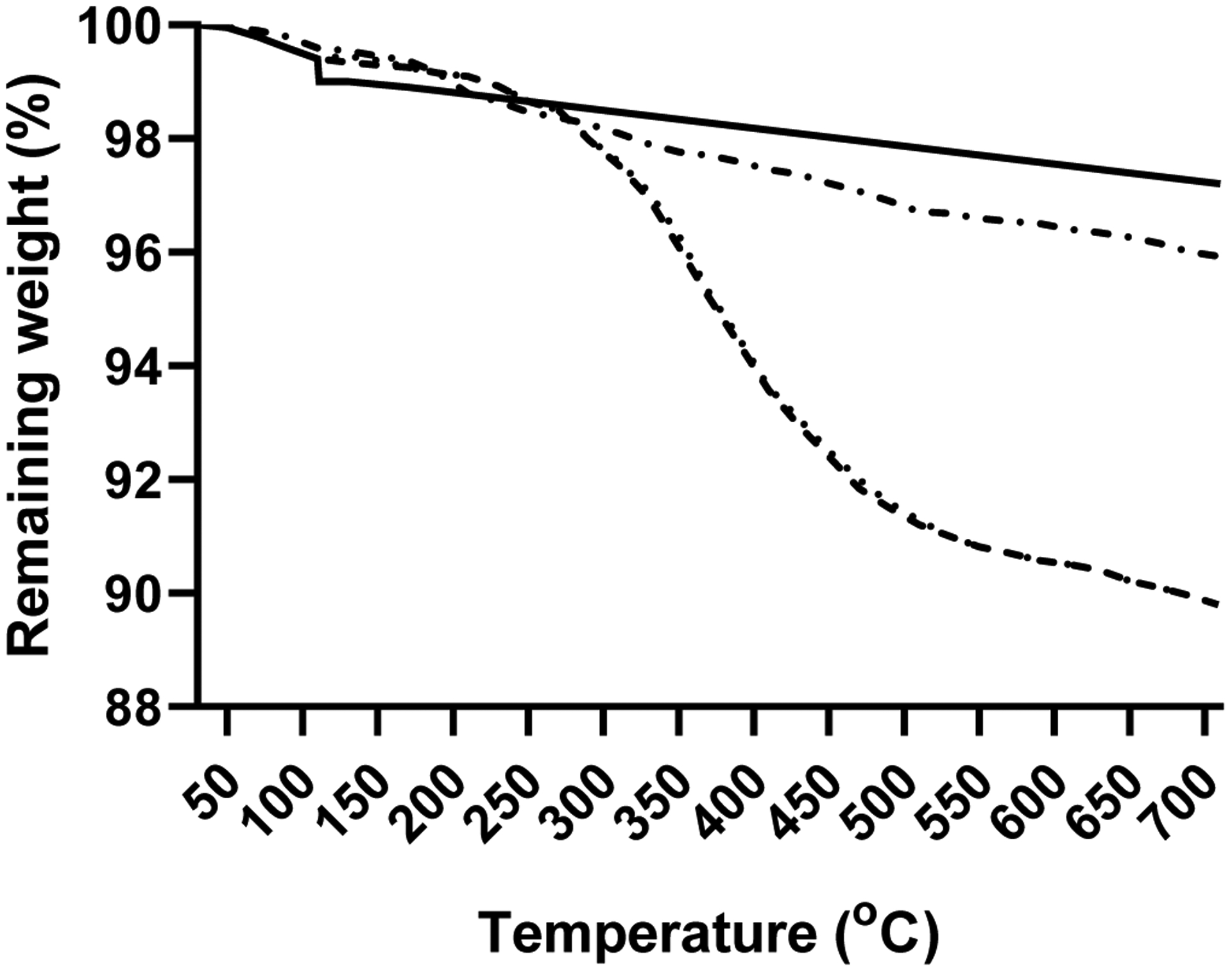
Thermogravimetric analysis curves of the as-synthesized nanoceria (solid line), nanoceria that had been through the procedure (dash-dot-dash line), rat plasma-incubated nanoceria(dotted line), and rat serum-incubated nanoceria (dashed line).

The nanoceria primary particle size averaged 12 (S.D. 2.9) nm, consistent with BET results of 71 m^2^/gm, which is equivalent to 11 nm. The particles were crystalline and polyhedral, with an isoelectric point of 3.0 [28]. The nanoceria hydrodynamic diameter before incubation with rat plasma or serum reveals considerable agglomeration of the as- prepared nanoceria in water, when compared to its primary particle size. Interaction with rat plasma or serum increased the nanoceria hydrodynamic size, perhaps due to protein adsorption (Figure 2). The hydrodynamic diameter distributions of the two serum replicates were not statistically different so the results were averaged. The two replicates of hydrodynamic diameters after plasma exposure had similar profiles. Although statistically different, we averaged the results. The nanoceria hydrodynamic diameter distributions after plasma or serum exposure were significantly different from each other and from the pre-exposure nanoceria distribution. The nanoceria citrate coating might be displaced by proteins [22] or remain on the surface and bind proteins [38]. Thermogravimetric analysis results show an ^~^3% weight loss of the as-prepared nanoceria attributed to water and other components and an additional ^~^1% loss after the nanoceria had been through the procedure in the absence of plasma or serum exposure (Figure 3). After incubation with rat plasma or serum, there was an additional ^~^6% weight loss over the temperature range of BSA weight loss [39] attributed to proteins coating the nanoceria. Nanoceria incubation with rat plasma or serum decreased the zeta potential (Table 1). The decrease in absolute zeta potential can be attributed to protein coating from plasma and serum.

**Figure 4:**
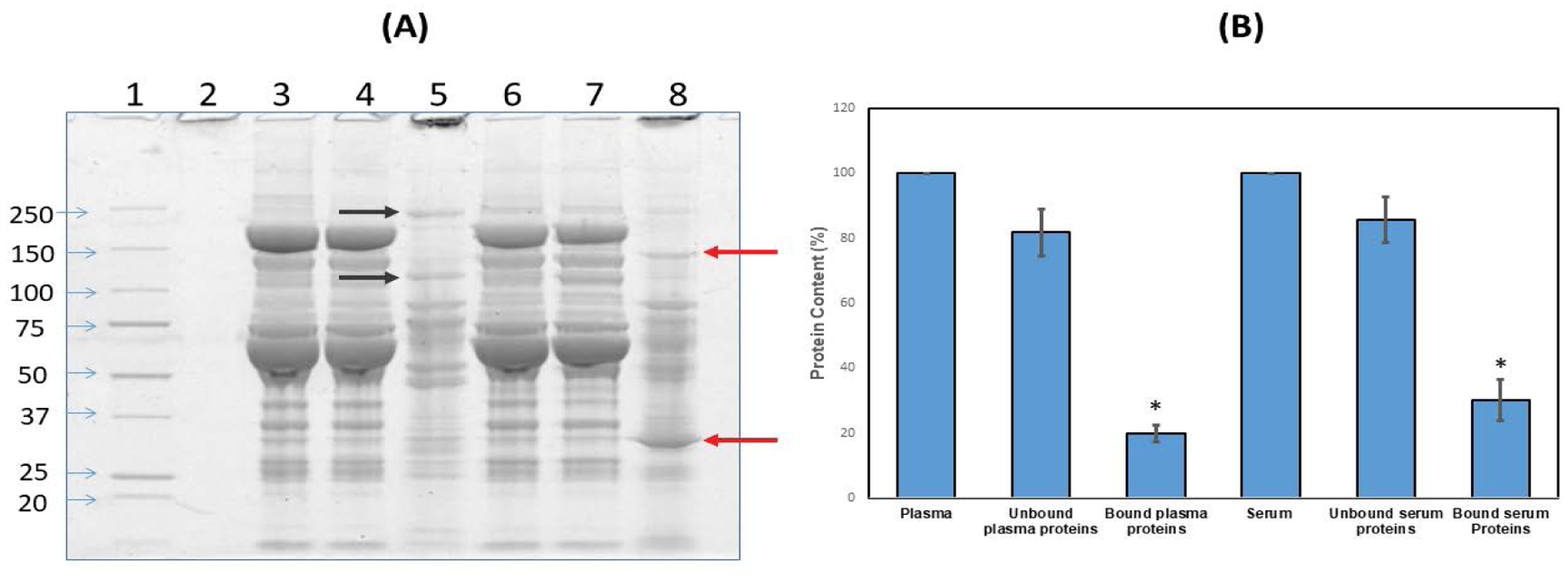
The protein profile of plasma and serum proteins bound to nanoceria. (**A**). Lane 1: Molecular weight markers, Lane 2: Nanoceria alone, Lane 3: Plasma, Lane 4: Unbound plasma proteins, Lane 5: Bound plasma proteins, Lane 6: Serum, Lane 7: Unbound serum proteins, Lane 8: Bound serum proteins. Arrows indicate bands with different proteins in plasma- vs. serum-incubated nanoceria samples. (**B**) ImageQuant image analysis of SDS-PAGE lanes 8 vs. 5 showed a greater percentage of the serum proteins interact with nanoceria than the plasma proteins. The gel image is representative of 4 independent experiments, for which the results are shown in B as mean ± S.D.

There have been many studies utilizing systemic and pulmonary nanoceria administration, the latter resulting in <1% of the nanoceria entering systemic circulation [40–42]. However, little is known as to what happens to nanoceria after it enters the circulatory system. In the present study, nanoceria incubated with plasma or serum led to increased protein size, shown as proteins that do not migrate into gels in contrast to plasma or serum alone, suggesting some nanoceria plasma and serum protein interaction (Figure 4A). The zeta potential decrease after plasma or serum exposure also suggests nanoceria-protein interaction. Zeta potential decrease during serum protein incubation, although over a much longer time, was shown [22]. We hypothesize that some of the plasma/serum proteins bound to nanoceria play a role in nanoceria agglomeration. To identify which plasma or serum proteins were associated with nanoceria, we employed proteomics.

Samples from the SDS-PAGE gel showed 1002 and 5648 ng cerium/mg gel at the top of lanes 5 and 8 (Figure 4A), respectively, but only 0.4 and 0.5 ng/mg gel in the center of those lanes, respectively, indicating that the separated proteins were essentially cerium-free. We treated the pellet with SDS-sample buffer followed by centrifugation and loading of the supernatant onto the gel. Based on our observation of intense staining at the top of the gel, we speculate that the speed we used for centrifugation did not pellet down all the ceria; some small nanoceria-protein complexes formed that have strong binding. When the proteins dissociated from the plasma/serum-incubated nanoceria were analyzed by SDS-PAGE, differences in protein profiles were observed, i.e., different bands and band intensities appear among proteins released from nanoceria that had been exposed to serum compared to plasma (Figure 4A arrows). Semi-quantitative densitometry analysis of total nanoceria-bound proteins showed that nanoceria incubated with serum samples had greater protein intensity than plasma-incubated nanoceria (Figure 4B). Since nanoceria incubation in plasma and serum was performed under identical conditions including addition of the same amount of proteins, differences in the protein profile observed on 1D-gel electrophoresis could be due to differences in the composition or amount of nanoceria-bound proteins. Serum is collected from blood after coagulation; therefore, some proteins involved in coagulation were removed that might lead to an enhanced ability of nanoceria to interact with other proteins. Table 2 shows proteins associated with nanoceria from either or both plasma and serum. Most of the proteins not eluted from serum-exposed nanoceria but eluted from plasma-exposed nanoceria are involved in blood coagulation. Interestingly, both plasma- and serum-incubated protein profiles showed a few strong bands as indicated by arrows in Figure 4A. This increased protein band intensity compared to the original plasma and serum samples suggests that nanoceria may preferentially and selectively bind these proteins, leading to their enrichment. Differential binding affinity of nanoceria for blood proteins has been shown. The binding affinity of fibrinogen with nanoceria was 40 pM whereas it was 37 nM with human serum albumin [25]. Nanoceria protein coating could affect its stability, distribution, and functional roles.

**Figure 5:**
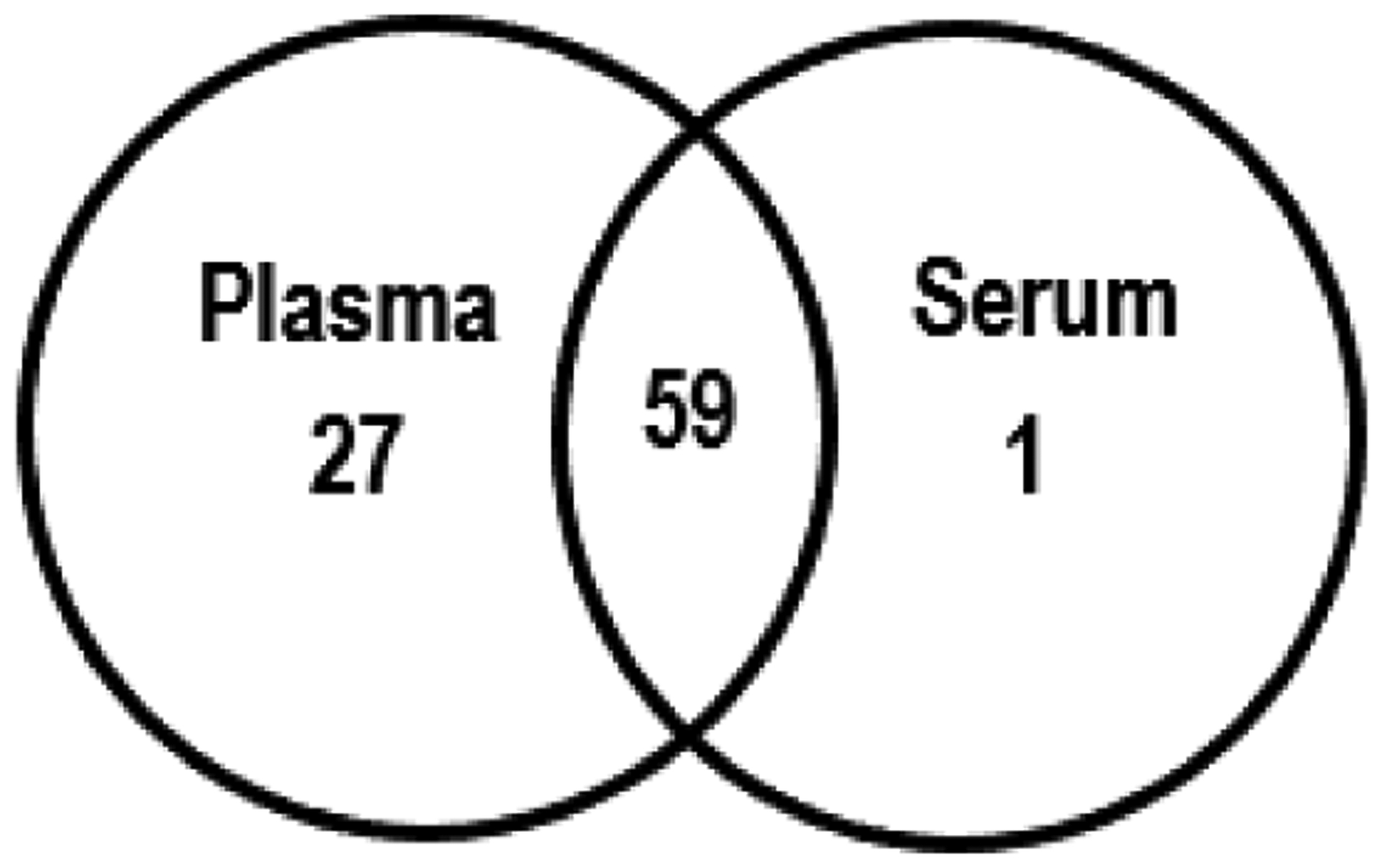
Venn diagram showing distribution of proteomics-identified, plasma or serum proteins associated with nanoceria.

To identify the proteins bound to nanoceria we subjected the proteins that had been bound to nanoceria to mass spectrometry-based proteomics. As can be seen from Table 2, most of the proteins that were identified from plasma- or serum-exposed nanoceria are common to both, suggesting that nanoceria might have selective preference to bind these proteins. A total of 87 proteins in plasma or serum interacted with nanoceria. Of these, 27 were unique plasma proteins, one was a unique serum protein, and 59 proteins in both plasma and serum associated with nanoceria (Figure 5). Further studies need to be conducted to understand the mechanisms underlying nanoceria’s preference to bind certain proteins and the resultant effects on both the proteins and nanoceria.

Coupled MS/MS and database interrogation-identified proteins bound to nanoceria were grouped into the following functional categories: antioxidant/detoxification; energy regulation; cell signaling; lipoprotein; complement pathways; immunoglobulin/immune function; blood coagulation; cellular iron homeostasis; proteolysis; inflammation, protein folding; protease inhibitors; carrier proteins; cell adhesion; protein/RNA degradation; and hormones (Table 2). Some comments about selected proteins within most of these categories follow:

### Antioxidant/detoxification:

The antioxidant activity of GPx3 depends upon its ability to convert lipid peroxides (or hydrogen peroxide) into the corresponding alcohol (or water), using glutathione as reducing equivalent. Since GPX plays a critical role as an antioxidant protein, its interaction with nanoceria conceivably could contribute to an altered cellular defense system.

### Glucose metabolism regulation:

GAPDH is not just an important enzyme of glycolytic pathway, which facilitates the enzymatic conversion of glyceraldehyde 3-phosphate to 1,3-biphosphoglycerate in glycolysis, but this enzyme also has other diverse functions [43–45]. Owing to its multiple isoforms and cellular localizations, GAPDH interacts with various small molecules, proteins and membranes, which are involved in normal as well as pathologic cellular functions including but not limited to transcription activation, apoptosis, and endocytosis [44,46]. Consequently, we speculate that binding of GAPDH to nanoceria conceivably might favor the transport of these particles via endocytosis processes into different cellular locations and into multiple organs.

### Cell signaling:

Phospholipase A2 belongs to family of phospholipase (PL) enzymes that hydrolyze phospholipids into fatty acids and other lipophilic substances. PLA2 cleaves the sn-2 acyl chain of phospholipids releasing unsaturated fatty acids, one of which is arachidonic acid, a lipid secondary messenger involved in cellular signaling and in inflammatory responses. Moreover, arachidonic acid is a major source of the lipid peroxidation product, 4-hydroxynonenal (HNE), which covalently binds proteins to change their structure and decrease their function [47]. If the binding of PLA2 to nanoceria negatively impacts the function of this PL, membrane integrity may be compromised and cell death processes promoted. On the other hand, if binding of PLA2 to nanoceria stabilizes this PL, this conceivably could contribute to several inflammatory diseases, including coronary artery disease [48], and acute respiratory distress syndrome [49], with both possibilities causing cellular damage.

### Lipoproteins:

Apolipoproteins (Apo) regulate the transport and distribution of lipids (including cholesterol in some cases) through the lymphatic and circulatory systems, serve as a cofactors or catalysts for lipid metabolic reactions, and maintain structure of lipoprotein particles. Moreover, ApoA1 is involved in regulating levels of the pro-inflammatory cytokine, TNFa [50], while plasma levels of ApoJ (clusterin) are correlated to protein aggregation and neurodegeneration [51]. Owing to their multiple functions, Apo regulate cellular lipoprotein metabolism [52]. Binding of lipoproteins to nanoceria might promote their transport or absorption to other organs such as liver and may affect its distribution and accumulation in the cells of different organs. Indeed, we previously showed that when nanoceria are administered systemically, liver accumulation is observed that is highly persistent and damaging to this critical organ [53]. Unexpectedly, following 90 days after systemic administration of nanoceria, we demonstrated that nanoceria are bio-transformed by the liver into different shapes and production of antioxidant Ce^3+^ [54], at a time that coincides with return to baseline of the elevated oxidative stress in brain [15]. ApoE, which is essential for the normal catabolism of trigyceride-rich lipoprotein constituents, also meditates the transport and uptake of cholesterol and lipid by interacting with different cellular receptors, including the low density lipoprotein (LDL) receptor.

### Complement pathways:

Complement pathways (CP) are an integral part of the innate immune system or non-specific immune response and they complement the antibiotic activity of antibodies in biological systems by augmenting the opsonization of bacteria by antibodies. CP can also be activated early in infection in the absence of antibodies. The interaction between nanoceria and the proteomics-identified proteins of the complement system could result from a binding affinity of these proteins to nanoceria, and this binding conceivably might activate this cellular defense system even in the absence of pathogenic insults.

### Immunoglobulin/immune function:

Immunoglobulins (Ig) are large, Y-shaped glycoproteins produced by B-cells and used by the immune system to identify and neutralize foreign objects such as bacteria and viruses. Interaction of Ig with nanoceria conceivably could materially affect these functions in a negative manner, potentially posing a risk to individuals who were treated with nanoceria-based antioxidant therapeutics as has been proposed [55].

### Blood coagulation:

As noted above, plasma levels of this category of proteins bound to nanoceria were more numerous identified by proteomics than those in serum, since such proteins were used to initiate blood clotting. Such a demarcation of binding of blood clotting proteins to nanoceria between plasma and ceria gives confidence that the proteomics methods employed give biologically relevant results.

### Iron homeostasis:

Transferrin is a key protein for binding iron ions. Adventitious iron is dangerous to cells, since Fe^2+^ is a pro-oxidant, converting hydrogen peroxide to hydroxyl free radicals via Fenton chemistry, which can lead to cell death [56]. Hence, binding loose iron ions is critical for cell survival. However, transferrin in both plasma and serum bind to nanoceria, thereby likely diminishing its iron-binding function and potentially posing a danger if nanoceria were used therapeutically.

### Proteolysis:

Plasma kallikrein is a serine protease that cleaves kininogen to produce the pro-inflammatory peptide, bradykinin. Hence, it is conceivable that binding of plasma kallikrein (not detected in serum) to nanoceria is protective by decreasing inflammatory processes.

### Protease inhibitors:

Unregulated activation of matrix metalloproteinases is associated with many disease states [57]. Metalloproteinase inhibitors contribute to the regulation of these enzymes [58]. Therefore, binding of metalloproteinase inhibitor-3 to nanoceria conceivably could weaken regulation of metalloproteinases, leading to nicks in endothelial tissues and consequent development of thrombosis. In contrast to high molecular weight kininogen, low molecular weight kininogen does not protect kallikrein from inactivation by C1 inhibitor. Therefore, adsorption of low molecular weight kininogen to nanoceria may be protective by preventing development of bradykinin-mediated inflammatory processes. Fetuin-B, an inhibitor of ovastacin and meprin-metalloproteinases, is suggested to be a potential contributor in proteinaceous networks involved in immune defense, extracellular matrix assembly, cell signaling, among other functions. Consequently, fetuin-B association with nanoceria could inhibit its function with implications for fibrosis, inflammation, cancer, and certain neurodegenerative disorders [59].

### Carrier/cargo proteins:

Hemopexin is a heme scavenging protein, thereby contributing to inhibition of heme-induced free radical formation. This protective effect is related to hemopexin-mediated induction of heme oxygenase-1 activity [60]. Vitamin D-binding protein complexes much of the vitamin D in plasma or serum. Decreases in vitamin D are associated with numerous clinical disorders, including Alzheimer disease [61]. Consequently, association of vitamin D- binding protein with nanoceria may make vitamin D less available for health.

## CONCLUSION

To our knowledge, this is the first proteomics study to identify plasma and serum proteins that coat nanoceria. One potential outcome of nanoceria protein coating is to enhance nanoceria uptake by cells. Nanoceria protein binding has been shown to alter protein structure. This may affect nanoceria’s pro- and/or anti-inflammatory properties. Here, consideration of various functional classes of proteins leads to the notion that, while many proteins adsorbed onto nanoceria would have negative consequences, some cellular effects would be more protective in nature by appropriate nanoceria binding. Hence, a better understanding of the interaction between nanoceria and plasma and serum proteins is essential. Protein binding might affect the function of nanoceria as well the functions of the proteins bound to them. As noted above, nanoceria has been proposed as a therapeutic agent, so it is our opinion that such uses are premature until critical evaluation of how nanoceria behave once in the body, especially in the blood and organs.

## Figures and Tables

**Table 1:** Nanoceria zeta potential before and after incubation with rat plasma and serum. Values are mean ± S.D.

Sample	Zeta potential (mV)
Nanoceria	−48
Rat plasma-exposed nanoceria	−28
Rat serum-exposed nanoceria	−20

**Table 2:** Proteins and their functional classification that were identified from rat plasma and/or serum bound to citrate-coated nanoceria.

Functions	Plasma	Serum
**1. Antioxidant/detoxification**		
Glutathione peroxidase 3	x	x
**2. Energy regulation**		
Creatine kinase M-type	x	x
Glyceraldehyde-3-phosphate dehydrogenase	x	x
Adenylate kinase isoenzyme 1	-	x
**3. Cell signaling**		
Phospholipase A1	x	-
Phospholipase A2	x	x
Insulin-like growth factor I	x	x
**4. Lipoproteins**		
Apolipoprotein A-I	x	x
Apolipoprotein A-II	x	x
Apolipoprotein A-IV	x	x
Apolipoprotein B-100	x	x
Apolipoprotein C-II	x	x
Apolipoprotein E	x	x
Apolipoprotein H	x	x
Apolipoprotein N	x	x
Apolipoprotein J (Clusterin)	x	-
**5. Complement pathways**		
Complement C3	x	x
Complement C4	x	x
Complement component C6	x	x
Complement component C8	x	x
Complement component C9	x	x
Complement component factor h-like 1	x	x
C4b-binding protein alpha chain	x	x
C4b-binding protein beta chain	x	x
Clusterin	x	-
Protein C4-2	x	x
Protein C8a	x	x
Protein Cfb	x	-
Protein Cfh	x	x
Protein F5	x	x
Protein Serpinf1	x	x
Mannose-binding protein A	x	x
**6. Immunoglobulins/immune function**		
Ig gamma-2A chain C region	x	x
Ig gamma-2B chain C region	x	x
Ig gamma-2C chain C region	x	x
Ig kappa chain C region, A allele	x	x
Macrophage stimulating 1	x	-
**7. Blood coagulation**		
Coagulation factor II, isoform CRA_a	x	x
Coagulation factor VII	x	x
Coagulation factor X	x	x
Coagulation factor XII	x	-
Coagulation factor XIII A chain	x	-
Coagulation factor XIII, beta subunit	x	-
Fibrinogen beta chain	x	x
Isoform Gamma-A of Fibrinogen gamma chain	x	x
Heparin cofactor 2	x	-
Carboxypeptidase B2	x	-
Carboxypeptidase N catalytic chain	x	-
Plasminogen	x	x
Platelet factor 4	x	-
Procollagen, type VI, alpha 3	x	x
Vitamin K-dependent protein C	x	x
Vitamin K-dependent protein S	x	x
Protein Mmrn1	x	-
Protein Serpinc1	x	-
Protein Serpinf1	x	x
Alpha-1-antiproteinase	x	x
CXC chemokine RTCK1	x	x
**8. Cellular iron homeostasis**		
Serotransferrin	x	x
Protein RGD1310507	x	-
Protein RGD1564614	x	-
**9. Proteolysis**		
Protein Serpina4	x	-
Plasma kallikrein	x	-
**10. Protein folding**		
78 kDa glucose-regulated protein	x	x
**11. Protease inhibitors (just inhibitors)**		
Alpha-1-macroglobulin	x	x
Glia-derived nexin	x	-
Isoform LMW of Kininogen-1	x	-
Inter alpha-trypsin inhibitor, heavy chain 4	x	x
Inter alpha-trypsin inhibitor, heavy chain 1	x	x
Metalloproteinase inhibitor 3	x	x
Serine protease inhibitor A3N	x	x
Protein AMBP	x	-
Serine protease inhibitor	x	-
Fetuin-B	x	-
**12. Carrier/cargo proteins**		
Alpha-2-HS-glycoprotein	x	x
Albumin	x	x
Hemopexin	x	x
Vitamin D-binding protein	x	-
Transthyretin	x	x
Retinol binding protein 4	x	x
**13. Cell adhesion/extra cellular matrix/structural**		
Anastellin	x	x
Extracellular matrix protein 1	x	-
Gelsolin	x	x
**14. Bone morphogenetic protein**		
Secreted phosphoprotein 24	x	-
**15. Protein/RNA degradation**		
Cullin-associated NEDD8-dissociated protein 1	x	x
**16. Hormone**		
Cystatin-related protein 1	x	-
**17. Unknown functions**		
Alpha-2-glycoprotein 1	x	x
Putative lysozyme C-2	x	-
